# RACIAL DIFFERENCES IN PREDICTORS OF CARDIOMETABOLIC RISK FOR OLDER US WOMEN

**DOI:** 10.1093/geroni/igae098.1367

**Published:** 2024-12-31

**Authors:** Christy Erving, K J Davidson-Turner, Cleothia Frazier, Jacquelyn Coats

**Affiliations:** The University of Texas Austin, Austin, Texas, United States; The University of Texas Austin, Austin, Texas, United States; The Pennsylvania State University, University Park, Pennsylvania, United States; Washington University in St. Louis, St. Louis, Missouri, United States

## Abstract

Though cardiovascular disease is the leading cause of death among women, significant racial disparities in cardiovascular disease persist. This study investigated psychosocial predictors of cardiometabolic risk (CMR), a count of high-risk cardiovascular and metabolic biomarkers, among older U.S women and whether predictors differed for Black, and Hispanic, and White respondents. Data were from the Health and Retirement Study (HRS; age 50 years+). Predictors including health behaviors, socioeconomic status, psychological factors, and relational demands (with spouse, children, relatives, friends) were drawn from the 2010/2012 waves. CMR, drawn from the 2014/2016 waves, was assessed using six biomarkers: pulse pressure, resting heart rate, C-reactive protein, waist glycosylated hemoglobin A1c, and total and high-density lipoprotein cholesterol. The sample comprised 6707 women (1197 Black, 672 Hispanic, 4838 White). Linear regression analyses were conducted to assess the association between the predictors and CMR in race-stratified models. Preliminary results revealed that predictors of CMR were distinct for different racialized groups. Specifically, for Black women, obesity and former smoking status were associated with increased CMR while working part-time was associated with decreased CMR. Among Hispanic women, having a GED or high school diploma (compared to less than a high school education) was associated with decreased CMR. Among White women, obesity, smoking, depressive symptoms, and having no spouse were associated with increased CMR while higher education, working part-time, and mastery were associated with decreased CMR. Surprisingly, no one factor was a predictor of CMR for all women; moreover, relational demands did not emerge as a significant predictor of CMR.

